# Cytokine and hormonal regulation of bone marrow immune cell Wnt10b expression

**DOI:** 10.1371/journal.pone.0181979

**Published:** 2017-08-11

**Authors:** Fraser L. Collins, Naiomy Deliz Rios-Arce, Laura R. McCabe, Narayanan Parameswaran

**Affiliations:** 1 Department of Physiology, Michigan State University, East Lansing, Michigan, United States of America; 2 Department of Radiology, Michigan State University, East Lansing, Michigan, United States of America; 3 Biomedical Imaging Research Centre, Michigan State University, East Lansing, Michigan, United States of America; Universite de Nantes, FRANCE

## Abstract

**Background & aims:**

Wnt10b is a crucial regulator of bone density through its ability to promote osteoblastogenesis. Parathyroid hormone has been shown to regulate Wnt10b expression in CD8^+^ T cells. However, the relative expression and other source(s) of Wnt10b in the bone marrow immune cells (BMICs) is unknown. Sex hormones and cytokines such as, estrogen and TNFα are critical regulators of bone physiology but whether they regulate BMIC Wnt10b expression is unclear. To determine the potential regulation of Wnt10b by estrogen and TNFα, we assessed Wnt10b expression by flow cytometry under estrogen- and TNFα-deficient conditions.

**Methods:**

Effects of TNFα was determined in male and female C57BL/6 wildtype and TNFα knockout mice. Effect of estrogen was investigated 4, 6 and 8 weeks post-surgery in ovariectomized Balb/c mice. Intracellular Wnt10b was detected using goat anti-mouse Wnt10b and a conjugated secondary antibody and analyzed by flow cytometry.

**Results:**

Wnt10b expression was sex- and lineage-specific. Females had 1.8-fold higher Wnt10b signal compared to males. Percent of Wnt10b^+^ myeloid cells was higher in females than males (8.9% Vs 5.4%) but Wnt10b^+^ lymphoid cells was higher in males than females (6.3% Vs 2.5%). TNFα ablation in males increased total BM Wnt10b expression 1.5-fold but significantly reduced the percentage of BM Wnt10b^+^ CD4^+^ T cells (65%), CD8^+^ T cells (59%), dendritic cells (59%), macrophages (56%) and granulocytes (52%). These effects of TNFα on Wnt10b were observed only in males. In contrast to TNFα, estrogen-deficiency had indirect effects on BMIC Wnt10b levels; reducing the average percentage of BM Wnt10b^+^ CD8^+^ T cells (25%) and granulocytes (26%) across an 8-week time course.

**Conclusion:**

Our results demonstrate unique cell type- and sex-dependent effects on BMIC Wnt10b expression. Together, our results reveal myeloid cells in the bone marrow as an important source of Wnt10b under complex hormonal and cytokine regulation.

## Introduction

Mesenchymal stem cells (MSCs) are pluripotent cells capable of differentiating into numerous cell types; including osteoblasts and adipocytes [1]. A key factor involved in determining MSC cell fate is the Wnt / β-catenin signaling pathway [2]. Of the Wnt proteins, Wnt10b is a critical regulator of osteoblast and adipocyte differentiation. Wnt10b-mediated signaling enhances osteogenesis through the induction of the transcription factors Runx2, Dlx5 and osterix while inhibiting adipogenesis, through suppression of the adipogenic transcription factors C/EBPα and PPARγ [3–6]. The importance of Wnt10b in osteoblast differentiation is highlighted in Wnt10b over-expressing mice which exhibit higher bone density and lower marrow adiposity compared to wild type mice [4]. Additionally, Wnt10b knockout mice have decreased trabecular bone due to a reduction in mesenchymal progenitor cells [7]. Thus, Wnt10b is a crucial player in bone homeostasis.

Numerous sources of Wnt10b in the bone marrow have been identified including immune cells, osteoblasts, osteoclasts, and adipocytes [3,8–10]. However, the contribution of these cell types to overall bone marrow Wnt10b levels remains unknown. Although osteoblasts are one of the primary sources of Wnt10b, work by the Pacifici group has demonstrated that Wnt10b gene expression is highly upregulated in CD8^+^ T cells in response to intermittent parathyroid hormone (iPTH) treatment. Their studies have also shown that lymphocyte-specific Wnt10b is required for maximal iPTH anabolic responsiveness [9]. Other studies have revealed elevated Wnt10b gene expression by TGFβ treated osteoclasts [10] and increased Wnt10b gene expression during bone marrow plasma cell differentiation [11,12]. However, the relative contribution of these and other immune cells to total bone marrow Wnt10b levels is not well-established. Furthermore, whether the expression of Wnt10b in various bone marrow immune cells is regulated under physiological and pathophysiological conditions is not fully known. Importantly, it is also not clear whether conditions that regulate immune cell Wnt10b do so by modulating the number of Wnt10b producing cells and/or by enhancing the expression of Wnt10b per cell.

Wnt10b expression is under the regulation of many hormones as well as inflammatory cytokines. While intermittent PTH has been shown to upregulate bone marrow Wnt10b gene expression the effect of estrogen is less obvious. In animal models of estrogen-deficiency (ovariectomy, OVX) loss of estrogen was shown to increase bone marrow (BM) T cell Wnt10b gene expression two weeks post-surgery [13]; a key step in OVX-induced expansion of hematopoietic stem and progenitor cells. Compared to the hormonal regulation of Wnt10b, tumor necrosis factor alpha (TNFα) has been shown to have dimorphic effects on Wnt10b expression [8,14,15]. TNFα transgenic mice display reduced Wnt10b and have lower bone density compared to wildtype mice [14,16] while treatment of osteoblast MC3T3-E1 cultures with TNFα significantly reduced Wnt10b gene expression [8,14]. This inhibition of Wnt10b expression by TNFα is thought to contribute to diminished osteoblast differentiation and increased osteoblast apoptosis observed in Type 1 diabetes. In contrast, treatment of human MSC cultures with TNFα increases Wnt10b gene expression subsequently increasing *in vitro* calcium deposition [15]. These data imply important and complex interactions between TNFα, Wnt10b signaling and regulation of bone density [8,14,17].

Given the important role of Wnt10b in the regulation of bone remodeling and the lack of information regarding the relative levels of Wnt10b in the various immune cells in the bone marrow, in the present study we examined regulation of Wnt10b levels in the bone marrow immune cells using flow cytometry. Our studies reveal unique cell type- and sex-dependent effects on Wnt10b expression in bone marrow immune cells as well as identify complex hormonal and cytokine-mediated effects.

## Materials and methods

### Animals and experimental design

Mice (B6.129S-*Tnf*^*tm1Gkl*^/J and their corresponding wild type breeder pairs) were obtained from The Jackson Laboratory (Bar Harbour, Maine). Mice were housed in a 12:12-h light-dark cycle at 23°C in groups of up to five animals per cage. Food (Teklad-2019; Envigo, USA) and water were given *ad libitum*. Knockout of TNFα (TNF^ko^) was confirmed by RT-PCR with primers specific for the wild type and knockout gene locus (based on The Jackson Laboratories protocol). Common primer was 5′ TAG CCA GGA GGG AGA ACA GA 3′, wild-type reverse primer was 5′AGT GCC TCT TCT GCC AGT TC 3′ and mutant reverse primer was 5′ CGT TGG CTA CCC GTG ATA TT 3′. PCR amplicons were separated on a 2% agarose DNA gel with a 100 bp DNA ladder (Invitrogen, Carlsbad, CA).

Female Balb/c mice 11 weeks of age were obtained from The Jackson Laboratory (Bar Harbour, Maine). Mice were allowed to acclimate to animal facility for 1 week prior to start of experiment. Mice were randomly split into two groups: sham control or OVX. For sham and OVX surgeries mice were placed under isofluorane anesthesia for < 10 minutes and a 2 cm lower-mid dorsal incision was made, extending through the skin and muscle layer. Ovaries were isolated and removed from the OVX cohort and incision sites closed using surgical staples. Mice were given Teklad 2019 chow (Madison, WI) and water *ad libitum* and were maintained on a 12 h light/dark cycle. Mice were sacrificed at 0, 4, 6 and 8 weeks post-surgery. All animal procedures were approved by the Michigan State University Institutional Animal Care and Use Committee and conformed to NIH guidelines.

### Wnt10b staining

Total bone marrow (BM), both nucleated and non-nucleated cells were isolated from the mouse femur. Specifically, femurs were isolated (specific sex and strain noted in individual studies and figure legends) and extraneous tissue/muscle detached. The femoral head was then severed and the femur placed cut side down into a 0.5 ml micro-centrifuge tube with a small hole in the base. The 0.5 ml micro-centrifuge tube was placed inside a 1.5 ml micro-centrifuge tube and centrifuged at 5000 rcf for 20 seconds. Bone marrow was collected in the 1.5 ml micro-centrifuge tube and re-suspended in 1 ml of alpha-MEM. Male, female, and ovx mice did not display differences in the total number of bone marrow cells isolated. Cells (@ 2x10^6^) were incubated with Fc block (BD Pharmingen, CA, USA) for 15 min. Cells were stained with anti-mouse CD3-AlexaFluor 700 (clone 500A2, eBioscience, CA, USA), anti-mouse CD4-V500 (Clone RM 4–5, BD Bioscience, CA, USA), anti-mouse CD8a-PE-Cyanine5.5 (clone 53–6.7, eBioscience), anti-mouse F4/80-APC AlexaFluor 780 (clone BM8, eBioscience) and anti-mouse CD11c-APC (clone N418, eBioscience) for 30 minutes at 4°C. Cells were washed three times in assay buffer (PBS, 0.5% bovine serum albumin (BSA), 5mM EDTA) followed by permeabilization in cytofix / cytoperm per manufacturer’s instructions (BD Biosciences). Intracellular staining was performed with either goat anti-mouse Wnt10b (Clone N-19; previously published for use in immunohistochemistry [18] and affinity chromatography [19]; Santa Cruz Biotechnology, Dallas, TX) or isotype control normal goat IgG (Santa Cruz Biotechnology) and incubated for 30 minutes at 4°C. Cells were washed and incubated with donkey anti-goat IgG-CFL 488 (Santa Cruz Biotechnology) for 30 minutes at 4°C before being analyzed by flow cytometry.

### Flow cytometry gating profile

Data were acquired on a BD LSRII (Becton Dickinson, Franklin Lakes, NJ) and analyzed with FlowJo (Version 10; FlowJo, LLC, Ashland OR). Doublets were removed by creating a singlet gate. T cells were determined by first gating on CD3^+^ cells then by gating on CD4^+^ or CD8^+^ cells. Macrophages were identified as being F4/80^+^ CD11c^+^. Dendritic cells were identified as CD11c^+^ F4/80^-^. Granulocytes (neutrophils and eosinophils) were gated according to their forward scatter (FSc) and side scatter (SSc) profiles. Wnt10b^+^ cell percentage was corrected for non-specific binding using the isotype control.

### Parathyroid hormone stimulation

Bone marrow was isolated from the femur of male C57BL/6 mice (15 weeks). To induce Wnt10b production, based on the study by Terauchi et al [9], 2x10^6^ cells were cultured in alpha-MEM, 10% fetal bovine serum (FBS) ± 50 nM parathyroid hormone (1–34) human (Sigma-Aldrich, St Lois, MO) for 3 hours at 37°C 5% CO_2_. Cells were stained for Wnt10b expression and analyzed by flow cytometry as described.

### TNFα stimulation

Bone marrow was isolated from the femora of TNF^ko^ male and female C57BL/6 mice (13–23 weeks) as described above. Cells (@ 2x10^6^) were plated in a 96 well U-bottom plate and cultured in alpha-MEM, 10% FBS ± TNFα (1 fg/ml to 50ng/ml). Cells were incubated at 37°C 5% CO_2_ for 24 hours. Plates were spun down at 1000 g for 5 minutes and supernatant removed before being washed twice in PBS. Cells were incubated in versene solution (Life Technologies, NY, USA) for 15 minutes to remove adherent cells before being stained and analyzed for Wnt10b expression as described.

### Statistical analysis

All measurements are presented as the mean ± SEM. Significant outliers were removed using the ROUT test for outliers. Student’s t-test and 1-way ANOVA were performed using GraphPad Prism software version 6 (GraphPad, San Diego, CA, USA). A *p*-value of less than or equal to 0.05 was considered significant.

## Results

### Detection of Wnt10b in bone marrow cells by flow cytometry

**Fig 1:**
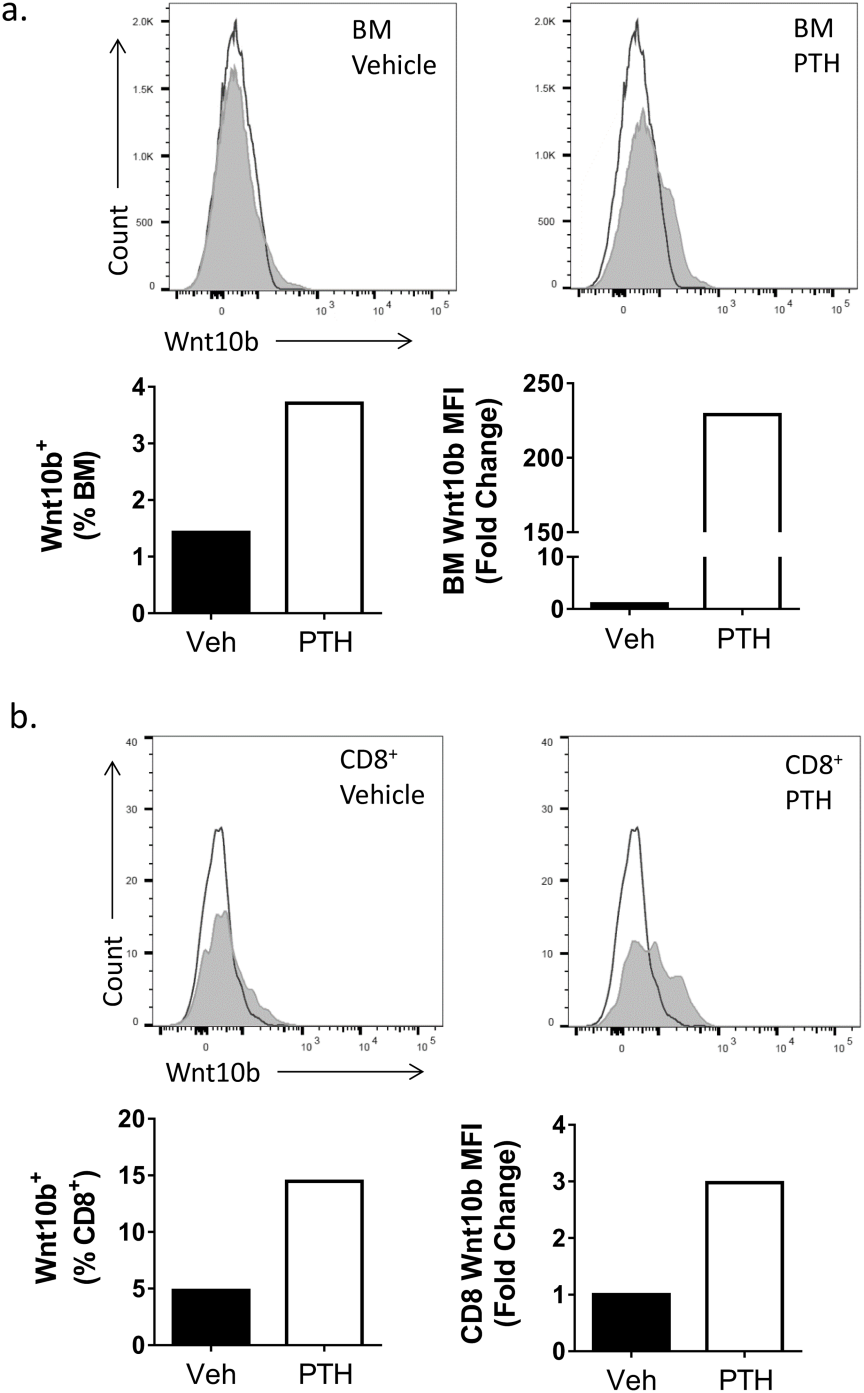
Detection of Wnt10b by flow cytometry. Bone marrow (BM) was isolated from the femur of a male C57BL/6 mouse (n = 1) and cultured ± 50nM PTH for 3 hours. Cells were stained for Wnt10b and analyzed by flow cytometry, gating on bone marrow and CD8^+^ T cells. In the bone marrow population (a) a shift in peak was observed for the Wnt10b stained cells over the isotype control corresponding to approx. 1.5% of vehicle treated cells. PTH increased the number of Wnt10b^+^ BM cells to 3.5% and the MFI greater than 200-fold. Gating on the CD8^+^ T cells (b) in the vehicle treated group identified approximately 5% were Wnt10b^+^. Following PTH treatment this increased 3-fold.

Expression of Wnt10b in bone marrow cells has previously been demonstrated at the mRNA level by qPCR [9]. To determine whether Wnt10b protein could be detected in cells by flow cytometry, bone marrow cells were isolated and stained using a goat anti-mouse Wnt10b antibody and a fluorescent-conjugated secondary antibody. A goat IgG isotype control was used to correct for non-specific binding. As a positive control, cells were stimulated with PTH, which has been demonstrated to significantly enhance bone marrow (BM) CD8^+^ T cell Wnt10b gene expression after 3 hours [9]. PTH stimulation led to ~3.5% Wnt10b^+^ bone marrow cells, compared to only ~1.5% in vehicle treated cells. In addition, PTH treatment resulted in a greater than 200-fold increase in Wnt10b median fluorescent intensity (MFI; Fig 1a). When BM cells were specifically gated on the CD3^+^ CD8^+^ T lymphocytes, a positive shift in the peak was identified for the Wnt10b antibody over the isotype control (~5% of the gated CD8^+^ T cells were also Wnt10b^+^). In the presence of PTH, Wnt10b^+^ CD8^+^ T cells increased to ~15%. This was accompanied by a 3-fold increase in Wnt10b expression (following PTH stimulation) in CD8 T cells as determined by Wnt10b MFI. Thus, PTH stimulation led to elevated bone marrow Wnt10b levels by increasing expression per cell as well as by increasing the percentage of cells expressing Wnt10b.

### Sex differences in bone marrow immune cell Wnt10b expression

**Fig 2:**
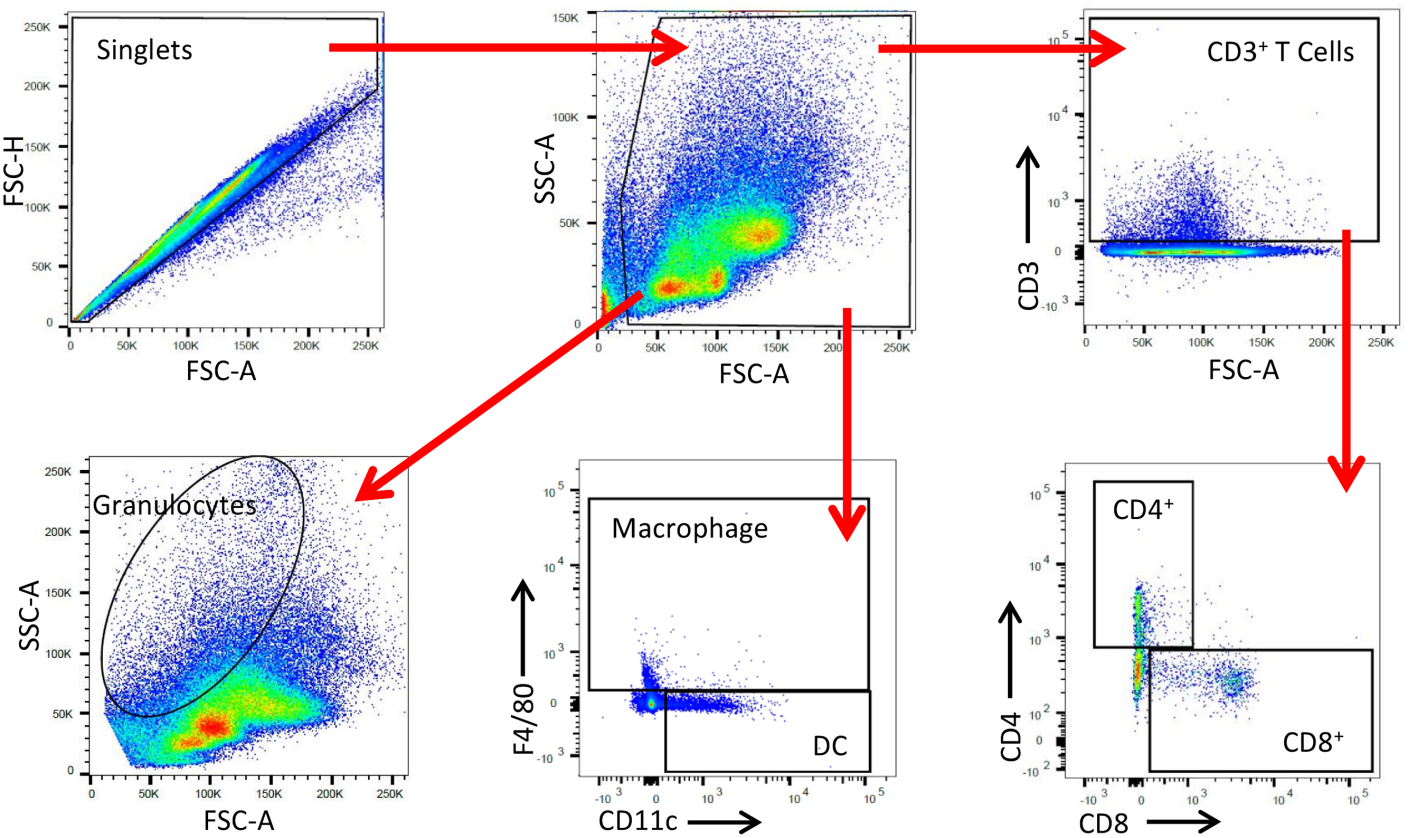
Wnt10b gating profile. Gating profile for analyzing Wnt10b expression in bone marrow immune cells. Doublets were removed by singlet gating and debris removed from analysis. T cells were selected by gating on CD3^+^ cells then further refined by gating on CD4^+^ and CD8^+^. Macrophages were gated as F4/80^+^ and F4/80^+^ CD11c^+^. Dendritic cells were classified as F4/80^-^ CD11c^+^. Granulocytes were gated according to their FSc and SSc.

**Fig 3:**
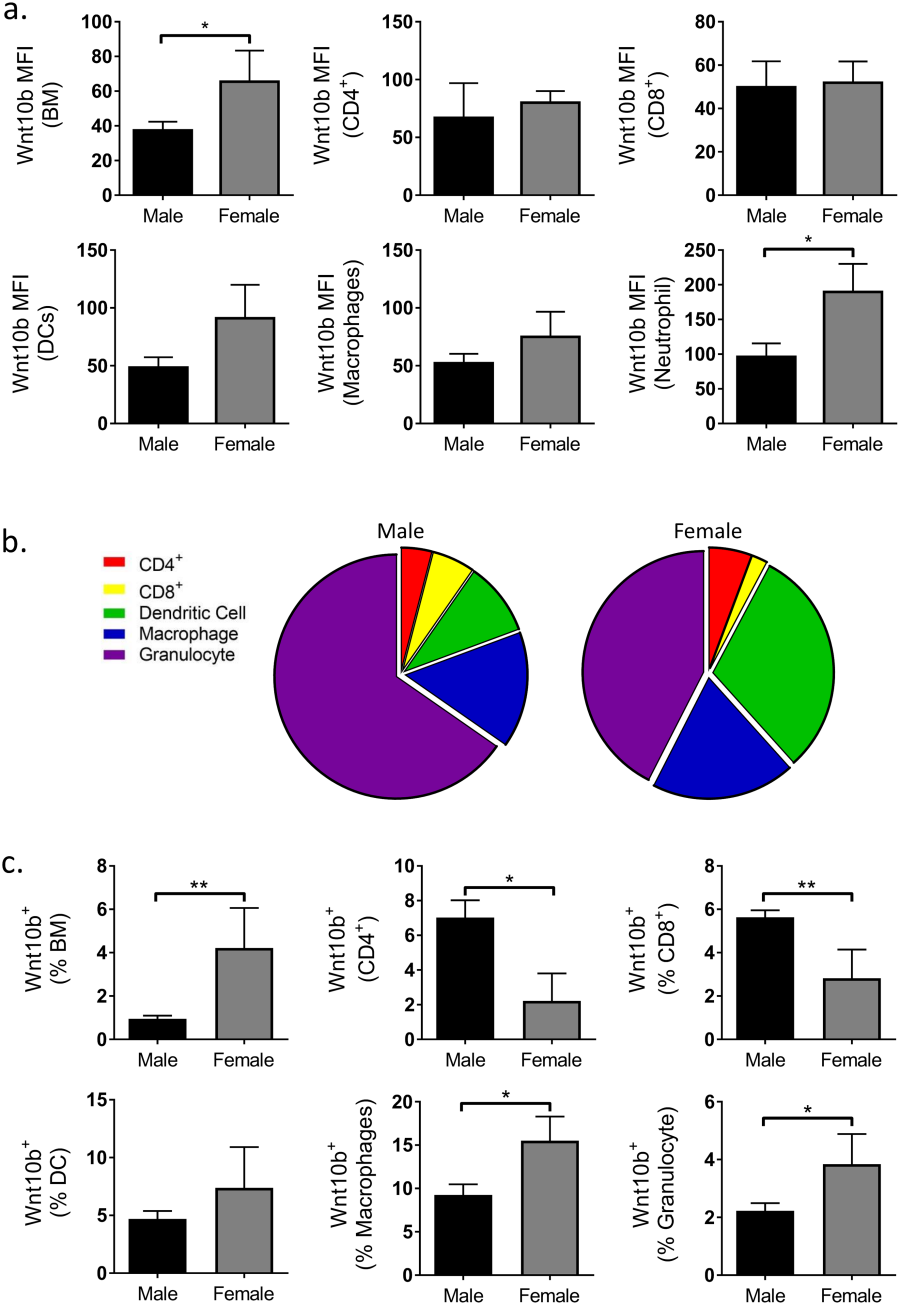
Comparison of male and female C57BL/6 bone marrow immune cell Wnt10b expression. Bone marrow (BM) was isolated from the femora of male (n = 22) and female (n = 5) C57BL/6 adult mice and analyzed by flow cytometry. Wnt10b MFI, % of cell type and % of total bone marrow was calculated. a) Female total BM expressed significantly higher levels of Wnt10b compared to males with significant cell-type specific sex-differences identified. b) Relative contribution of cell-types investigated to the Wnt10b^+^ population in males and females. c) Female total BM had a higher % of Wnt10b^+^ cells compared to males; cell-type specific differences in Wnt10b expression profiles were observed between the sexes. Statistical analysis performed by student’s t-test; *p<0.05 and **p<0.01.

Having demonstrated that expression of Wnt10b could be reliably detected by flow cytometry we next sought to examine immune cell Wnt10b expression profiles in male and female mice. Bone marrow cell populations were gated and defined as in Fig 2. Analysis of MFI identified a significantly higher Wnt10b signal in the female BM compared to males (*p*<0.045). This could be attributed to higher granulocyte as well as DC Wnt10b signal in the female mice (Fig 3a).

In addition to differences in Wnt10b expression per cell, the percentage of Wnt10b^+^ cells was also different between males and females. Significantly higher proportions of total BM Wnt10b^+^ cells in females (4.2±1.8%) were observed compared to males (0.9±0.1%; *p*<0.003). Further gating of the bone marrow cells identified cell-type specific differences in the expression profiles of Wnt10b between males and females (Fig 3b and 3c). In particular, comparison of myeloid lineage cells revealed that the percentage of macrophage and granulocyte Wnt10b^+^ cells was significantly elevated in female compared to male mice (macrophage: 9.3±1.2% Vs 15.5±2.8%; *p*<0.04 and granulocyte: 2.2±0.3% Vs 3.8±1.0%; *p*<0.04). No difference was observed for dendritic cells. When taken as a percentage of the total bone marrow however, Wnt10b^+^ macrophages (0.19±0.03% Vs 0.37±0.10%; *p*<0.03) as well as dendritic cells (0.12±0.02% Vs 0.59±0.22%; *p*<0.0002) were significantly higher in the females (Table 1). No difference was observed between sexes for granulocyte Wnt10b expression. Note however, that while the proportion of macrophages was similar between males and females, dendritic cells were higher and granulocytes lower in the females compared to male bone marrow (Table 1). These differences could account for the variations between the sexes in terms of Wnt10b expression when analyzed as a percent of the respective cell population versus percent of the total bone marrow cells.

**Table 1:** Comparison of male and female Wnt10b^+^ cells as percentage of total bone marrow.

Cell Type	Male (% Wnt10b^+^)	Female (% Wnt10b^+^)
Total BM	0.95 ± 0.15	**4.2 ± 1.9** **
CD4^+^	0.05 ± 0.01	0.11 ± 0.07
CD8^+^	0.07 ± 0.01	**0.04 ± 0.02** *
DC	0.12 ± 0.02	**0.59 ± 0.22** **
Macrophage	0.19 ± 0.03	**0.37 ± 0.1** *
Granulocyte	0.81 ± 0.14	0.82 ± 0.37

Male and Female C57BL/6 Wnt10b^+^ Cells as a % of the Total Bone Marrow. Values are averages ± SEM. Male n = 22; female n = 5.

* = *p*<0.05;

** = *p*<0.01.

In contrast to myeloid cells, CD8^+^ Wnt10b^+^ T cells were significantly higher in males compared to females when analyzed as a percent of CD3^+^ T cells (5.6±0.3% Vs 2.8±1.3%; *p*<0.005) as well as a percent of total bone marrow cells (0.07±0.01% Vs 0.04±0.02%; *p*<0.04). Although CD4^+^ Wnt10b^+^ cells were higher in males compared to females when expressed as a percent of CD3^+^ T cells (7.0±1.0% Vs 2.2±1.6%; *p*<0.04), this difference was lost when expressed as a percent of the total BM cells. This likely was due to a modest increase in CD4^+^ T cell percent in the female BM (Table 2). Note that the proportion of CD8^+^ T cells was modestly *lower* in the females compared to males (Table 2). Taken together, these results demonstrate that even though males have significantly higher percentage of CD8^+^ Wnt10b^+^ cells in the BM, females have a significantly higher percent macrophages and dendritic cells that are Wnt10b^+^ and have higher total Wnt10b^+^ BM cells.

**Table 2:** Cell type as percentage of C57BL/6 bone marrow.

Cell Type	Male		Female	
	WT	TNF^ko^	WT	TNF^ko^
CD4^+^	0.79±0.11	0.52±0.09	0.95±0.20	0.65±0.03
CD8^+^	1.03±0.05	0.77±0.09*	0.93±0.2	1.03±0.1
DC	3.0±0.1	3.1±0.2	**5.3±0.4**^##^	4.2±0.3
Macrophage	3.1±0.4	2.3±0.1	3.5±0.6	4.9±0.5
Granulocyte	30.0±1.4	31.4±2.5	**17.9±2.9**^##^	12.4±0.9

Male and Female C57BL/6 WT and TNF^ko^ Bone Marrow Cell Percentages. Values are averages ± SEM. Male n = 22; female n = 5.

* = *p*<0.05 compared to male WT;

^##^ = *p*<0.01 compared to male WT.

### TNFα regulates bone marrow immune cell Wnt10b expression

**Fig 4:**
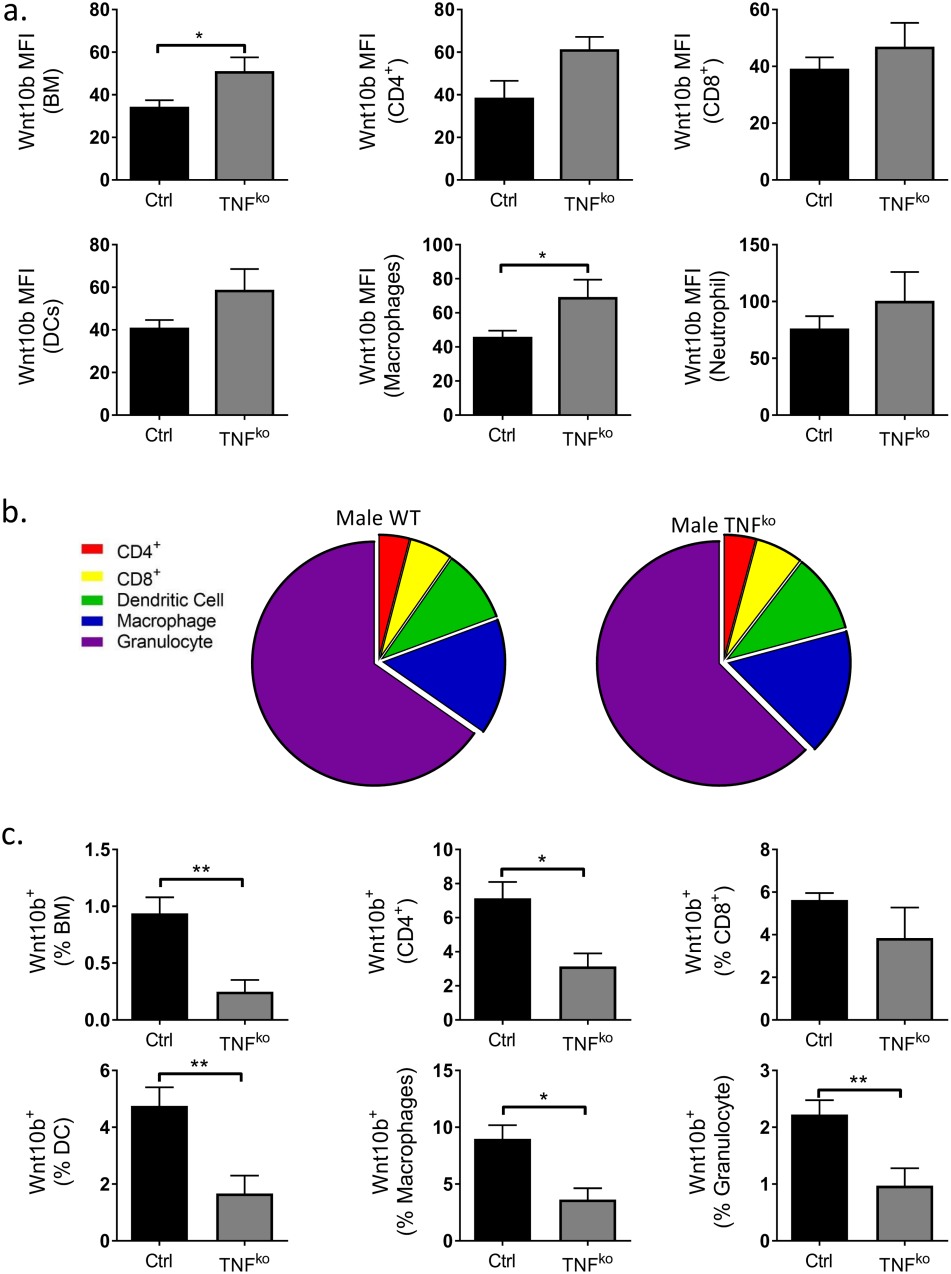
Regulation of male C57BL/6 bone marrow Wnt10b expression by TNFα. Bone marrow (BM) was isolated from the femora of male WT (n = 23) and TNF^ko^ (n = 9) C57BL/6 mice and analyzed by flow cytometry. Wnt10b MFI, % of cell type and % of total bone marrow was calculated. a) Ablation of TNFα significantly increased male BM and BM immune cell Wnt10b cellular expression. b) Relative contribution of cell-types investigated to the Wnt10b^+^ population in male WT and TNF^ko^ mice. c) Numbers of Wnt10b^+^ cells as a % of cell type and total BM were significantly reduced in the TNF^ko^ mice compared to WT. Statistical analysis performed by student’s t-test; *p<0.05 and **p<0.01.

Previous studies have demonstrated that TNFα stimulation decreases osteoblast Wnt10b gene expression [8,14]. Therefore, we hypothesized that ablation of TNFα would lead to increased BM immune cell Wnt10b. To test this, we compared Wnt10b expression of C57BL/6 TNFα knockout (TNF^KO^) and wild type (WT) mice. As predicted, in males, BM Wnt10b expression (as assessed by MFI) was significantly increased in TNF^KO^ compared to WT cells (*p*<0.04). A similar increase was specifically observed in macrophages as well as a modest increase in CD4^+^ T cell and DCs (Fig 4a).

Further gating of the bone marrow cells revealed that the relative contribution of each cell-type investigated to the Wnt10b^+^ population was comparable between the WT and TNF^ko^ (Fig 4b). Surprisingly however, the percent of BM Wnt10b^+^ cells in male TNF^KO^ mice was significantly decreased (3.8 fold; *p*<0.004) compared to male WT mice (Fig 4). Correspondingly, TNF^KO^ BM exhibited significant decreases in Wnt10b^+^ cells in both lymphoid and myeloid compartments and this was the case when analyzed as percent of the respective cell population as well as relative to the total BM cells (Table 3). This was observed despite a significant decrease in the percent of CD8^+^ T cells in male TNF^KO^ mouse BM (Table 2).

**Table 3:** Comparison of male and TNF^ko^ Wnt10b^+^ cells as percentage of total bone marrow.

Cell Type	Male	
	WT (% Wnt10b^+^)	TNF^ko^ (% Wnt10b^+^)
CD4^+^	0.05 ± 0.01	0.02 ± 0.01*
CD8^+^	0.07 ± 0.01	0.03 ± 0.01**
DC	0.12 ± 0.02	0.05 ± 0.02*
Macrophage	0.19 ± 0.03	0.08 ± 0.02*
Granulocyte	0.81 ± 0.14	0.30 ± 0.11*

Male C57BL/6 WT and TNF^ko^ Wnt10b^+^ Cells as a % of the Total Bone Marrow. Values are averages ± SEM. WT n = 23; TNF^ko^ n = 9.

* = *p*<0.05;

** = *p*<0.01.

**Fig 5:**
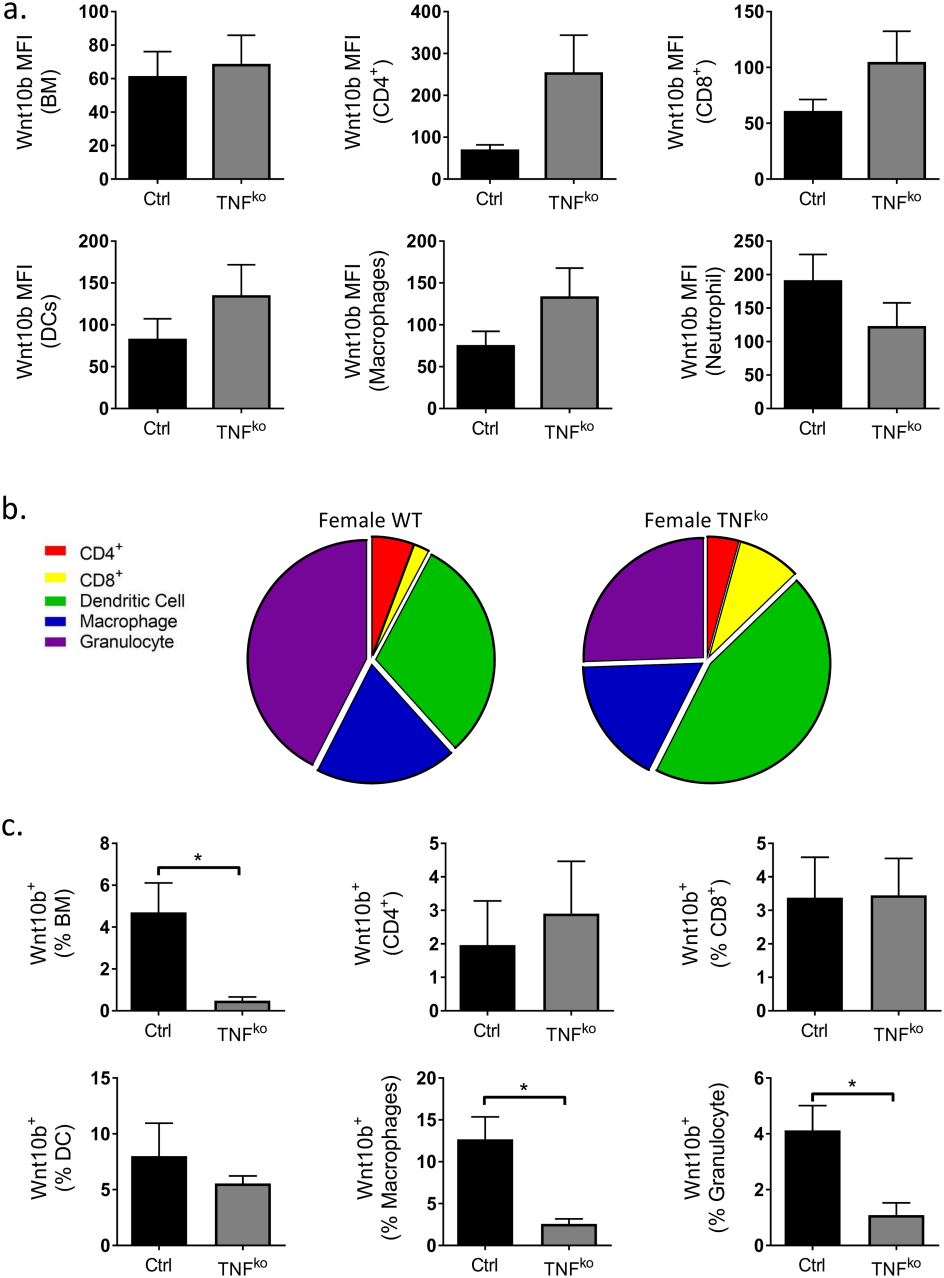
Regulation of female C57BL/6 bone marrow Wnt10b expression by TNFα. Bone marrow (BM) was isolated from the femora of female WT (n = 7) and TNF^ko^ (n = 5) C57BL/6 mice and analyzed by flow cytometry. Wnt10b MFI, % of cell type and % of total bone marrow was calculated. a) Loss of TNFα resulted in modest increases in BM immune cell Wnt10b expression. b) Relative contribution of cell-types investigated to the Wnt10b^+^ population in female WT and TNF^ko^ mice. c) Female TNF^ko^ exhibited a significant decrease in BM Wnt10b^+^ cells, Wnt10b^+^ macrophages and granulocytes. Statistical analysis performed by student’s t-test; *p<0.05 and **p<0.01.

In contrast to the male mice, loss of TNFα had no effect on overall total BM Wnt10b expression in females; expression per cell trended to increase in CD4^+^ and CD8^+^ T cells, DCs and macrophages but was offset by a decrease in granulocytes (Fig 5a). Furthermore, the relative contribution of each cell-type investigated to the Wnt10b^+^ population in females differed between the WT and TNF^ko^ (Fig 5b). Similar to male mice however, ablation of TNFα in females (Fig 5c) resulted in a significant decrease (*9*.*8 fold; p*<0.03) in total BM Wnt10b^+^ cells. The cell-specific changes in Wnt10b expression were found to have similarities and differences compared to the male mice. Similar to male mice, the proportion of Wnt10b^+^ macrophages (*4*.*9-fold*; *p*<0.01) and granulocytes (*3*.*8-fold*; *p*<0.02) was significantly decreased in TNF^KO^ compared to WT cells. In contrast to males, the % of Wnt10b^+^ CD4^+^ T cells and DCs was not altered in TNF^KO^ female mice. When analyzed as a percentage of the total BM population Wnt10b expression generally trended to be lower in the TNF^KO^ BM, however the results were too variable to make statistical conclusions (Table 4). Taken together these results reveal that endogenous TNFα decreases Wnt10b expression in specific immune cells in a sex-specific manner. However, TNFα appears to be equally important for determining the proportion of Wnt10b^+^ cells such that ablation of TNFα markedly decreases Wnt10b^+^ cells in the bone marrow in both males and females.

**Table 4:** Comparison of female WT and TNF^ko^ Wnt10b^+^ cells as percentage of total bone marrow.

Cell Type	Female	
	WT (% Wnt10b^+^)	TNF^ko^ (% Wnt10b^+^)
CD4^+^	0.09 ± 0.05	0.02 ± 0.01
CD8^+^	0.03 ± 0.01	0.04 ± 0.02
DC	0.63 ± 0.18	0.21 ± 0.03
Macrophage	0.30 ± 0.09	0.08 ± 0.01
Granulocyte	1.29 ± 0.58	0.12 ± 0.04

Female C57BL/6 WT and TNF^ko^ Wnt10b^+^ Cells as a % of the Total Bone Marrow. Values are averages ± SEM. WT n = 7; TNF^ko^ n = 5.

### Effect of TNFα on bone marrow Wnt10b in TNFα KO mice

**Fig 6:**
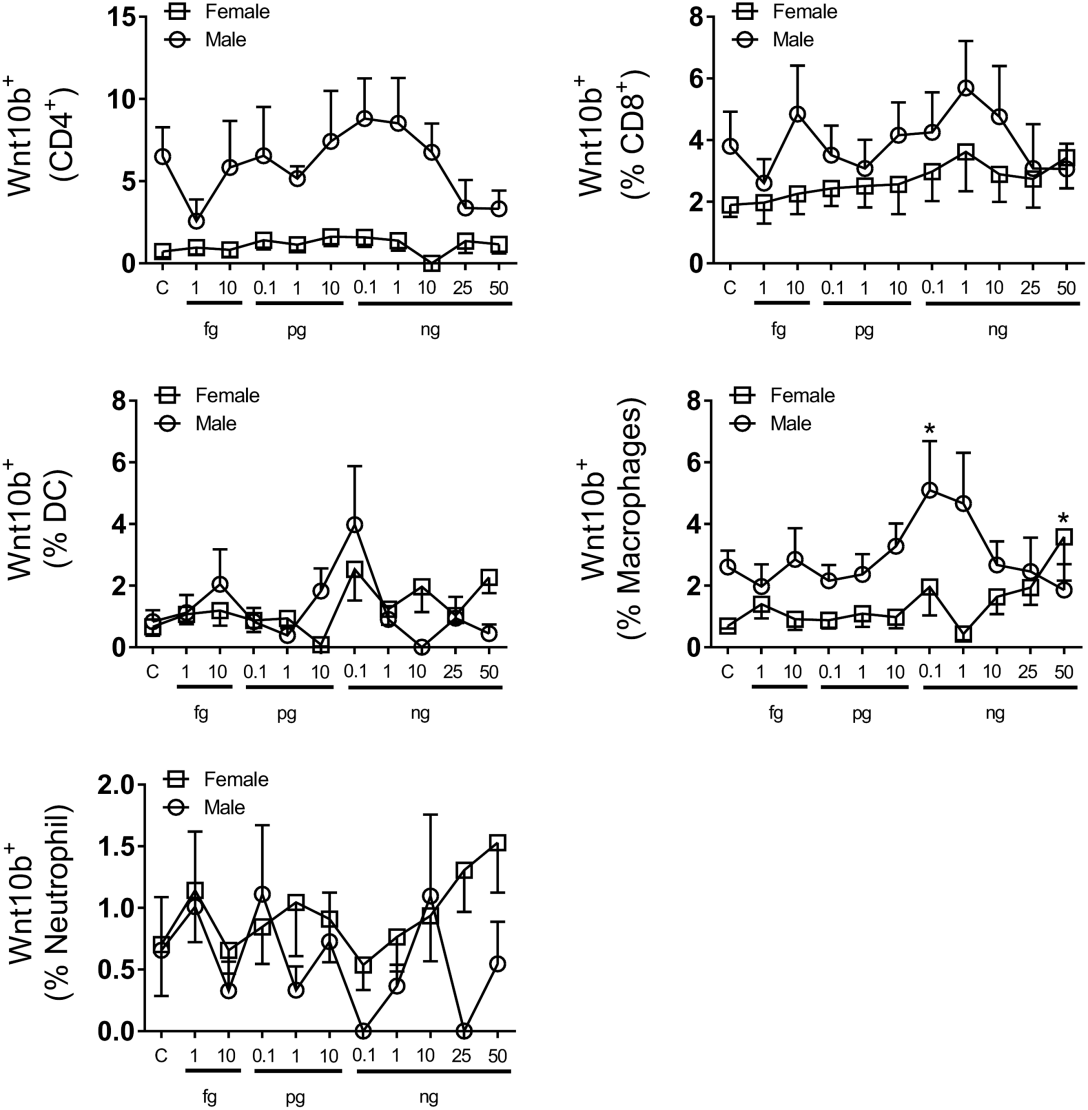
Effect of TNFα on C57BL/6 bone marrow Wnt10b in TNFα KO mice. Bone marrow (BM) was isolated from the femora of male TNF^ko^ (n = 5–7) and female TNF^ko^ (n = 9) C57BL/6 mice and cultured in the presence of TNFα (1 fg/ml– 50 ng/ml) for 24 hours before being analyzed by flow cytometry for Wnt10b. TNFα add-back modulated numbers of Wnt10b^+^ cells in male BM cultures; significantly increasing Wnt10b^+^ macrophages at 0.1 ng/ml. No significant effect on Wnt10b^+^ cell number was observed in female BM culture following addition TNFα with the exception of macrophages at 50 ng/ml. Statistical analysis performed by 1 Way ANOVA with Fisher’s LSD post-test; *p<0.05.

Having identified that TNFα expression is linked *in vivo* to Wnt10b expression we next sought to determine whether addition of TNFα to bone marrow cells isolated from the TNF^KO^ mice could restore expression (Fig 6). TNFα add-back to BM isolated from male mice resulted in a concentration-dependent response. In both CD4^+^ and CD8^+^ T cells very low levels of TNFα (0.1 fg/ml) resulted in a modest decrease in Wnt10b^+^ cells. Physiological levels of TNFα (1 pg/ml– 0.1 ng/ml [20]) resulted in a trend towards increased Wnt10b^+^ cells in CD4^+^ and CD8^+^ T cells. In contrast, concentrations higher than 1 ng/ml, representative of an inflammatory response, reduced Wnt10b levels. In macrophages, addition of TNFα at 0.1 ng/ml significantly increased Wnt10b^+^ cell numbers compared to the control. As with T cells, levels of TNFα above 1 ng/ml resulted in decreased Wnt10b expression. No changes in Wnt10b expression were observed in dendritic cells or granulocytes. Analysis of Wnt10b in female KO mice demonstrated that CD4^+^ and CD8^+^ cells had no significant response or trends to add-back of TNFα, which is in contrast to BM isolated from male KO mice. However, a significant increase in macrophage Wnt10b^+^ cells was observed at 50 ng/ml TNFα, though no response was seen at lower doses. These results suggest sex differences and highlight that in some cases deficiency of TNFα may be directly linked to a decrease in Wnt10b^+^ cells while in other cases the effect on Wnt10b^+^ cell numbers *in vivo* may be indirect.

### Estrogen-deficiency significantly reduces bone marrow immune cell Wnt10b expression

**Fig 7:**
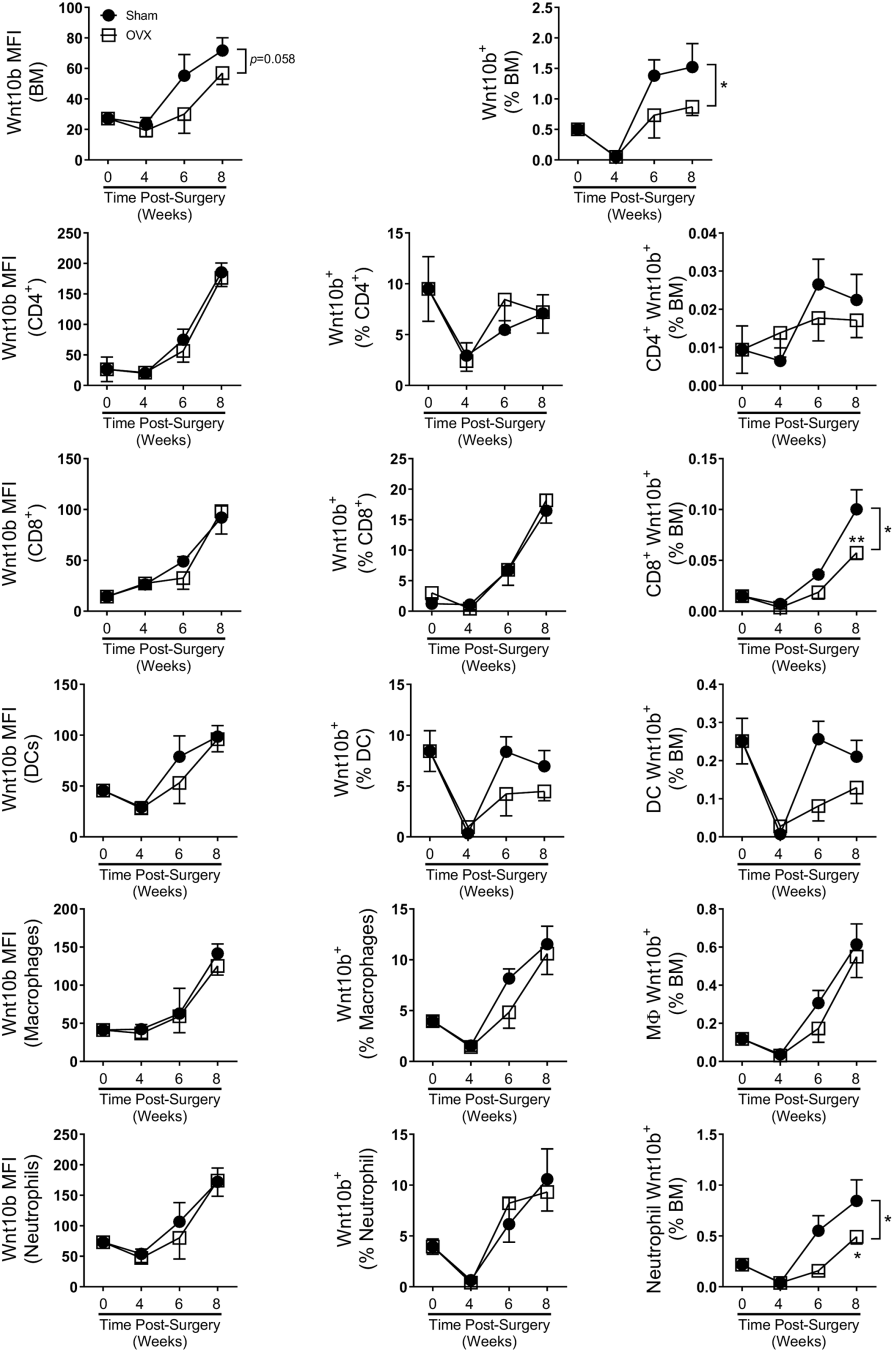
Effect of estrogen-deficiency on Balb/c bone marrow Wnt10b expression. Female Balb/c mice (12 weeks) underwent either sham or ovariectomy (OVX) surgery. Mice were harvested at 4 (n = 13), 6 (n = 4) or 8 (n = 10) weeks and bone marrow (BM) isolated from a femur and analyzed by flow cytometry. Wnt10b MFI, % of cell type and % of total bone marrow was calculated. OVX lowered total BM Wnt10b cellular expression but had no effect on any of the cell types investigated. % of Wnt10^+^ cells were significantly reduced in the OVX cohort across the time course compared to the sham control. Statistical analysis performed by 2-way ANOVA with Sidak post- test; *p<0.05.

To test if estrogen deficiency modifies Wnt10b immune cell profiles, we analyzed cells isolated from sham and OVX mice 4, 6 and 8 weeks post-surgery (Fig 7). Comparison of bone marrow cellular phenotype between sham and OVX mice revealed that OVX mice had significantly reduced the percentage of CD4^+^ T cells (*p*<0.02), CD8^+^ T cells (*p*<0.001), dendritic cells (*p*<0.0001) and granulocytes (*p*<0.004) across the time-course (Table 5) consistent with previous reports [21,22]. The total bone marrow Wnt10b signal (MFI) was reduced by OVX across the time course compared to the sham (*p*<0.058). However, no differences in Wnt10b signal intensity were observed in any of the specific cell types investigated, suggesting that estrogen-deficiency could affect Wnt10b expression in other bone marrow cells not included in the present study. Subsequent analyses of Wnt10b^+^ cells indicated significantly reduced levels of BM Wnt10b^+^ cells across the experimental time-course in the OVX mice (ANOVA *p*<0.04), with a reduction in the percentage of Wnt10b positive cells observed at 6 and 8 weeks. Further gating of the bone marrow cells revealed cell-type specific responses to loss of estrogen. As a percent of the total bone marrow cells, there was a significant decrease in Wnt10b^+^ CD8 T cells and granulocytes and a modest decrease in Wnt10b^+^ DCs in the OVX bone marrow compared to shams, especially at 6 and 8 weeks after surgery. Together, these results demonstrate that estrogen deficiency differentially regulates the proportion of Wnt10b^+^ immune cells in the bone marrow.

**Table 5:** Cell type as percentage of Balb/c bone marrow.

Cell Type	4 Week		6 Week		8 Week		OVX Effect
	Sham	OVX	Sham	OVX	Sham	OVX	
CD4^+^	0.58±0.08	0.44±0.03	0.48±0.09	0.31±0.04	0.29±0.03	0.23±0.02	*p*<0.05
CD8^+^	0.56±0.07	0.35±0.05**	0.56±0.07	0.33±0.00	0.59±0.04	0.49±0.05	*p*<0.01
DC	2.5±0.1	2.0±0.1**	3.3±0.1	2.6±0.2	3.1±0.1	2.6±0.2*	*p*<0.01
Macrophage	3.4±0.3	3.4±0.20	3.6±0.5	3.6±0.4	5.3±0.3	5.3±0.4	
Granulocyte	6.8±0.9	5.9±1.2	10.6±1.9	5.9±0.5*	8.6±0.4	5.7±0.5	*p*<0.01

Sham and OVX Balb/c Bone Marrow Cell Percentages. Values are averages ± SEM. 4 weeks n = 13; 6 weeks n = 4; 8 weeks n = 10.

* = *p*<0.05 significant to corresponding sham control;

** = *p*<0.01 significant to corresponding sham control.

## Discussion

Wnt10b is a critical regulator of bone formation, driving osteogenesis and inhibiting adipogenesis [3–6]. While previous studies have identified bone marrow cellular sources of Wnt10b, the relative contribution of the cell types remains unclear as the methods used (qPCR, western blot and immunohistochemistry) do not allow for comparative analysis [8,9]. Understanding the cell-type specific regulation of Wnt10b expression is important because of the location of the bone marrow cells and the role of Wnt10b in regulating osteogenesis. In the current study, we used Wnt10b antibody to detect intracellular Wnt10b in bone marrow cells and determine the number of Wnt10b expressing cells as well as the relative level of Wnt10b per cell. We used PTH as a positive control for the flow cytometry method since it is known to significantly induce Wnt10b gene expression in bone marrow CD8^+^ T cells after 3 hours stimulation [9]. Consistent with previous gene expression studies, we found that PTH increased bone marrow Wnt10b MFI signal and the number of positive cells over baseline. Furthermore, the data revealed that the antibody combination used was sensitive enough for the detection of Wnt10b at baseline as well as stimulated conditions by flow cytometry.

We report here that female C57BL/6 mice exhibit increased Wnt10b expression and increased percentage of Wnt10b positive cells compared to males. One possible explanation for this sex-specific difference could be that at the age used (13–23 weeks) female mice exhibit increased levels of bone remodeling [23]. Thus increased Wnt10b expression would be required to initiate osteoblast differentiation and bone formation. Breakdown of the bone marrow immune cell Wnt10b expression profile highlighted further remarkable sex-differences. Male C57BL/6 mice were found to have a higher percentage of CD8^+^ Wnt10b^+^ cells compared to the females. In contrast, females had higher percentages of Wnt10b^+^ DCs and macrophages. Previous studies have suggested that BM T cells are a critical source of Wnt10b, specifically following estrogen-deficiency [9,13]. In our studies, under baseline conditions however, granulocytes were found to contribute the largest percent of Wnt10b^+^ cells in both males and females. Indeed, CD8^+^ Wnt10b^+^ cells accounted for less than 0.1% of BM cells. This data suggests that under normal physiological conditions BM T cells might potentially play a relatively smaller role in total Wnt10b expression. This is further supported by a study in which Roser-Page et al [24] showed that only anergic CD8^+^ T-cells are a significant source of Wnt10b. Under basal conditions anergic CD8^+^ T cells are rare and so would likely not be a significant source of Wnt10b.

In our studies, estrogen-deficiency did not have any significant effect on Wnt10b expression in any of the cell types investigated. This is in concurrence with a study by D’Amelio et al [25], who demonstrated that there was no difference in Wnt10b gene expression in peripheral blood nucleated cells isolated from healthy controls and osteoporotic women. Interestingly however, in our model, Wnt10b expression was found to be reduced in the total BM across the time course. This suggests that estrogen does potentially modulate Wnt10b expression in some cell types, though it is not clear from this study what those cells are. In contrast however, Li et al [13], showed Wnt10b gene expression to be upregulated in BM T cells two weeks post-ovariectomy. These apparent discrepancies could be due to different strains of mice used (Balb/c in our versus C57BL/6 in Li et al [13]) as well as the time after OVX surgery. Balb/c and C57BL/6 mice respond to OVX differently with variations in trabecular and cortical bone loss [26]. In addition, it is possible that soon after loss of estrogen BM T cell Wnt10b expression is upregulated (as seen by Li et al) initially to stimulate bone formation to try and compensate for the increased bone resorption. As the mice enter a ‘late menopause-like’ stage and levels of osteoclastic bone resorption drop, Wnt10b expression may decrease [27]. In the present study, while loss of estrogen did not affect cellular Wnt10b expression, the percent of Wnt10b^+^ cells in the BM was indeed affected. When analyzed as a percentage of the total bone marrow, both CD8^+^ T cells and granulocytes contributed to reduced Wnt10b^+^ cells. This is due to reduced numbers of the cells being present in the OVX mice across the time-course. Although OVX has previously been shown to reduce BM neutrophil numbers in mice [21] the effect on CD8^+^ T cells is less clear. Previous studies in C57BL/6 mice have shown that OVX induces T cell proliferation and decreases T cell apoptosis [28]; increases T cell activation [29]; or has no effect on total T cell numbers [22]. In contrast, a study in 6 month-old female Sprague-Dawley rats revealed that OVX reduced bone marrow CD8^+^ T cell numbers over a 28 day time period [30]. These potential discrepancies in CD8^+^ T cell response to OVX may be due to the different strains used or to differences in analysis; with some studies reporting the data as a % of the bone marrow and others converting to total cell number.

Studies suggest that TNFα has a potential biphasic effect on Wnt10b, both enhancing and suppressing gene expression [8,14,15]. Addition of 1 ng/ml TNFα to cultures of human MSCs or 2–10 ng/ml TNFα to cultures of the murine pre-adipocyte 3T3-L1 cell line significantly increased Wnt10b gene expression [15,31,32]. However, in murine MC3T3-E1 osteoblast cultures 10 ng/ml TNFα was sufficient to inhibit Wnt10b expression [8]. Analysis of stroma-derived cell lines obtained from long-term BM cultures from TNF-deficient mice revealed significantly increased Wnt10b gene expression compared to wild-type mice [33]. Our current study adds to the growing body of evidence for a complex biphasic role of TNFα on Wnt10b expression. Deletion of TNFα increased cellular Wnt10b expression but decreased the percentage of BM Wnt10b^+^ cells. This suggests that within the immune cells there are sub-populations that are differentially regulated by TNFα for Wnt10b expression. The biphasic role of TNFα and sex-specific responses on Wnt10b expression was further supported by the TNFα add-back experiments. In BM cells from males, increasing levels of TNFα resulted in increased Wnt10b expression up to a certain point where high levels of TNFα then inhibited Wnt10b. In contrast, however, BM-derived from female mice showed no significant pattern to TNFα add-back.

In summary the results from the present study demonstrate for the first time that BM immune cell Wnt10b expression is different between males and females in C57BL/6 mice and highlights the relative contribution of each immune cell type in the context of the total bone marrow population. We further reveal that TNFα has sex-specific effects on Wnt10b expression and acts directly on specific BM immune cell sub-populations in a biphasic manner to induce or inhibit Wnt10b expression. Furthermore, we demonstrate that while loss of estrogen does not directly affect immune cell Wnt10b expression it indirectly reduces BM Wnt10b^+^ immune cell numbers over time via modulation of numbers of specific cell types. These finding provide new insight on the complexities of Wnt10b regulation.
